# Development of serum glycosylated exosomal microRNAs as biomarkers for early diagnosis of lung adenocarcinoma

**DOI:** 10.3389/fmed.2025.1695874

**Published:** 2025-12-10

**Authors:** Zhixin Huang, Jing Chen, Qi Gao, Kun Hao, Chundong Yu, Yi Huang

**Affiliations:** 1 Department of Clinical Laboratory, Fujian Children’s Hospital (Fujian Branch of Shanghai Children's Medical Center), Fuzhou, China; 2 Department of Clinical Laboratory, Fuzhou First General Hospital Affiliated with Fujian Medical University, Fuzhou, China; 3 Research and Development Center, Beijing Youngen Technology Co. Ltd., Beijing, China; 4 Research and Development Center, Beijing Hotgen Biotech Co. Ltd., Beijing, China; 5 State Key Laboratory of Cellular Stress Biology, Innovation Center for Cell Signaling Network, School of Life Sciences, Xiamen University, Xiamen, China; 6 Center for Experimental Research in Clinical Medicine, Fujian Provincial Hospital, Fuzhou, China; 7 Department of Clinical Laboratory, Fujian Provincial Hospital, Fuzhou, China; 8 Central Laboratory, Fujian Provincial Hospital, Fuzhou, China; 9 Fujian Provincial Key Laboratory of Cardiovascular Disease, Fujian Provincial Key Laboratory of Critical Care Medicine, Fuzhou, China

**Keywords:** lung adenocarcinoma, GlyExo-Capture approach, serum exosomal miRNA, early diagnosis, biomarkers

## Abstract

Early diagnosis is a major challenge in lung adenocarcinoma (LUAD). Tumor-derived exosomal microRNAs (miRNAs) are promising diagnostic biomarkers, and given the aberrant overexpression of tumor-associated glycans on exosomes, we employed a GlyExo-Capture approach using wheat germ agglutinin (WGA)- and lentil lectin (LCA)-coated magnetic beads to enrich glycosylated exosomes (WGA- and LCA-exosomes) from serum, and then detected exosomal miRNAs in 413 serum samples. Initially, small RNA sequencing was performed on a screening set (*n* = 30) to obtain candidate WGA-exosomal miRNAs. Moreover, candidate WGA-exosomal miRNAs were identified through RT-qPCR to develop a predictive panel of candidate WGA-exosomal miRNAs combined with candidate LCA-exosomal miRNAs identified in our pilot study via an independent training set (*n* = 254). Finally, the diagnostic value of the predictive panel for early LUAD was determined through a validation set (*n* = 129). Results showed that WGA- and LCA-coated magnetic beads effectively enriched glycosylated exosomes from both the conditioned media of LUAD cells and the sera of LUAD patients. Furthermore, a 4-miRNA panel of serum WGA-exosomal miR-199a-3p, miR-222-3p, combined with serum LCA-exosomal miR-486-5p, miR-139-3p, was developed for early diagnosis of LUAD. In the training and validation sets, the area under the curve of the 4-miRNA panel was 0.909 and 0.942, respectively. These findings suggest that the serum glycosylated exosomal 4-miRNA panel developed using the GlyExo-Capture approach may serve as a promising strategy for liquid biopsy-based early detection of LUAD.

## Graphical abstract

**Figure:**
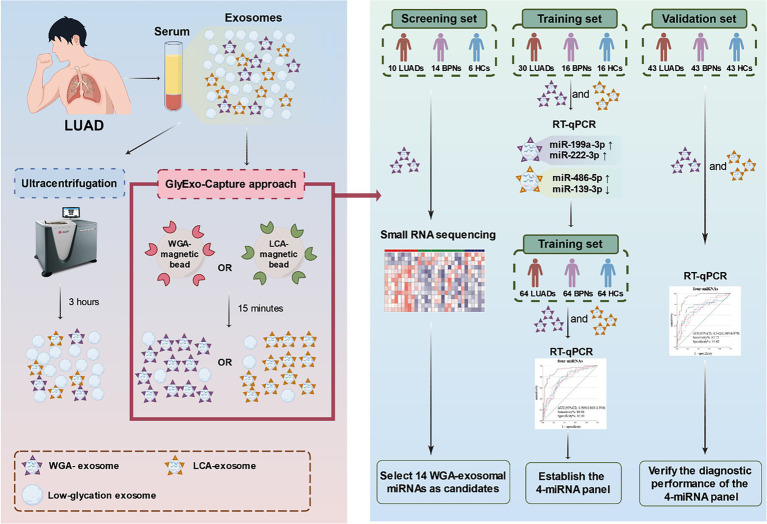

## 1 Background

Lung cancer has significantly contributed to cancer-related mortality, posing a severe threat to global human health (1), of which lung adenocarcinoma (LUAD) is the prevalent histologic subtype with 35–40% of all cases in recent years (2). Currently, surgical resection is still the most effective treatment for LUAD. However, due to the lack of obvious symptoms in early stages, most patients with LUAD face the diagnosis until the late stage of the disease, rendering the 5-year overall survival rate only around 15% (3). Given this, early diagnosis and timely surgical intervention are paramount for improving LUAD prognosis.

Exosomes, a specific type of extracellular vesicle (EV) ranging in size from 40 to 200 nm (4), are released by cells into the extracellular environment and have been demonstrated to be responsible for the transport of various cargoes from cells, such as proteins, RNA, and DNA (5). By transferring these cargoes, exosomes play a critical role in mediating intercellular communication and influencing a wide range of physiological and pathological processes. Notably, exosomes possess a protective lipid bilayer membrane that shields their cargoes from enzymatic degradation and external environmental stressors, such as temperature fluctuations or oxidative conditions. This structural integrity ensures the stability of their cargoes, both *in vivo* and during *in vitro* storage and handling (6, 7), making exosomes a promising tool for liquid biopsy, particularly in the early detection of cancer.

Among these cargoes, microRNAs (miRNAs) are particularly significant due to their stability and regulatory roles, and they have been well-documented as being enriched in exosomes derived from tumor cells (8). These miRNAs play a crucial role in the occurrence and development of a variety of malignancies by regulating tumor cell behaviors, angiogenesis, microenvironment, etc. (9–11). Importantly, exosomal miRNAs have shown great potential as non-invasive biomarkers for lung cancer diagnosis. For instance, Wu et al. (12) found that hsa-miR-103b, hsa-miR-877-5p, and hsa-miR-29c-5p were significantly upregulated in plasma exosomes of LUAD patients. Another study reported that serum exosomal miR-128-3p and miR-33a-5p could serve as a circulating biomarker for NSCLC (13). Additionally, a study established a 4-miRNA exosomal panel, consisting of miR-106b-3p, miR-125a-5p, miR-3615, and miR-450b-5p, as a key diagnostic biomarker for LUAD (14). Given that tumor-derived exosomal miRNAs reflect the biological information of their origin cells, we infer that they hold greater potential for specific tumor diagnostics compared to total serum exosomal miRNAs, which include contributions from normal cells. However, since tumor-derived exosomes constitute only a small fraction of the total exosomes in the serum, achieving ultra-specific identification and isolation is critical to advancing exosome-based miRNA diagnostics for LUAD.

Aberrant glycosylation is a key feature in tumor development (15, 16). Common glycosylation modifications, such as fucosylation, N-acetylglucosamine (GlcNAc), and sialylation, are frequently overexpressed on glycoproteins and glycolipids present on the surface of tumor cells and tumor-derived exosomes, contributing to malignancy (17–20). Numerous studies have shown that lectins such as lentil lectin (LCA) and wheat germ agglutinin (WGA) exhibit well-defined glycan-binding specificities: LCA predominantly recognizes core-fucosylated N-glycans (21), whereas WGA primarily binds GlcNAc and, to a lesser extent, sialylated motifs (22). Leveraging this, we developed a GlyExo-Capture approach using LCA- and WGA-coated magnetic beads to enrich tumor-derived glycosylated exosomes. In our pilot study, LCA-coated magnetic beads enriched LCA-exosomes for RT-qPCR detection, identifying a 4-miRNA panel that differentiated LUAD patients from benign pulmonary nodules (BPNs) and healthy controls (HCs) (AUC: 0.8554, sensitivity: 91.07%, specificity: 66.36%) (23). In this study, we also used WGA-coated magnetic beads to capture exosomes with GlcNAc and sialylation. We observed that WGA-coated and LCA-coated magnetic beads efficiently enriched glycosylated exosomes (WGA-exosomes and LCA-exosomes) from LUAD cell conditioned media (CM) and the sera of LUAD patients, providing a practical strategy for preferential isolation of tumor-associated exosomal populations in LUAD.

In this study, we first performed small RNA sequencing to identify candidate serum WGA-exosomal miRNAs. Subsequently, we validated these candidate WGA-exosomal miRNAs for early LUAD detection through RT-qPCR and developed a predictive panel that includes WGA-exosomal miR-199a-3p and miR-222-3p, along with LCA-exosomal miR-486-5p and miR-139-3p, identified in our pilot study, using an independent training set. Encouragingly, in an independent validation set, we confirmed the optimal diagnostic potential of a 4-miRNA panel comprising WGA- and LCA-exosomes for identifying early-stage LUAD patients.

## 2 Materials and methods

### 2.1 Patients and clinical samples

The retrospective cohort included serum samples from 147 early-stage LUAD patients, 137 BPN patients, and 129 HCs, collected from Fujian Provincial Hospital from October 2022 to June 2023. These samples were allocated at random to screening, training, and validation sets (Supplementary material Table S1). All patients conformed to the diagnostic criteria endorsed by international or professional associations, had no other comorbidities, and had not received any standard-of-care treatment prior to serum collection. Serum samples were uniformly processed under standardized conditions, such as blood collection, centrifugation, −80 °C storage, and consistent pre-analysis storage duration. Our study was approved by the Institutional Review Board of Fujian Provincial Hospital, and written informed consent was obtained from all participants.

### 2.2 Cell culture

The human LUAD cell lines PC-9 and H1299 and the normal cell lines BEAS-2B and NL-20 were obtained from ATCC and cultured in 5 mL of their respective media: RPMI-1640 for PC-9 and H1299, DMEM for BEAS-2B, and Ham’s F-12 for NL-20, each supplemented with 1% fetal bovine serum (Procell, catalog #164210). All cells were incubated at 37 °C with 5% CO₂ at saturated humidity for 72 h, after which the CM was collected for further analysis. Cell line authentication by STR profiling was performed in June 2023, and all cells were confirmed to be mycoplasma-free.

### 2.3 Exosome extraction by GlyExo-Capture approach

First, 700 μL of cell CM or 250 μL of serum was added to 500 μL of either WGA- or LCA-coupled magnetic bead solution (MBL) (Hotgen Biotech, catalog #723913 for WGA and #723914 for LCA) in an EP tube and incubated for 1 min. Next, the tube is placed on a magnetic rack (Hotgen Biotech, catalog #21390) for 1 min to separate the magnetic bead–exosome complex from the supernatant, which is then discarded. Subsequently, a wash buffer (WBL) (Hotgen Biotech, catalog #723920) was introduced into the tube, placed on the magnetic rack, and the supernatant was removed. Finally, the sample was then mixed with 250 μL of elution buffer (EBL) (Hotgen Biotech, catalog #723930) and incubated for 1 min, followed by the collection of the supernatant for storage.

### 2.4 Exosome extraction by ultracentrifugation (UC)

Approximately 13,000 μL of cell CM or 750 μL of serum was first centrifuged at 500 *g*, followed by centrifugation at 2000 *g*. Subsequently, the supernatant was further centrifuged at 10,000 *g* for 30 min, followed by filtration through a 0.22-μm sterile filter. Finally, ultracentrifugation at 120,000 *g* was performed, and the supernatant was carefully removed. This ultracentrifugation step was repeated once, followed by resuspension in 250 μL phosphate-buffered saline (PBS) for subsequent experiments.

### 2.5 Transmission electron microscopy (TEM)

Approximately 5–10 μL of exosome solution (pre-fixed with 2.5% glutaraldehyde (China National Pharmaceutical Group, catalog #30092436)) was added onto a copper grid and adsorbed for approximately 5 min. Then it was stained with 10 μL saturated uranyl acetate solution (China National Pharmaceutical Group, catalog #SPI-02624) for 1 min and subsequently incubated with ddH2O for 5 min twice. Observation was conducted under a JEM1400 TEM with an operating voltage of 80 kV and a magnification scale of 100 nm.

### 2.6 Nanoparticle tracking analysis (NTA)

Exosome samples were diluted with PBS to achieve a final concentration of approximately 1 × 10^8^–1 × 10^9^ particles/mL, which is recommended for optimal NTA measurement. A total of 500 μL of the diluted suspension was loaded into the NanoSight NS300 sample chamber under identical instrument settings for all measurements. The system was configured with the following settings for tracking and measurement: the laser wavelength was set to 520 nm and the filter wavelength was set to detect scatter. The camera operated at a frame rate of 60 frames/s, with a gain of 24.0 and a shutter speed of 70.000070. The capture duration for each measurement cycle was optimized based on the sample concentration and particle size to ensure accurate data acquisition. Data analysis was performed using ZetaView software (version 8.05.14 SP7).

### 2.7 Western Blot (WB) assay

Samples were lysed on ice with protein lysis buffer and the concentration was determined by bicinchoninic acid (BCA). Equal amounts of exosomal protein (20 μg per lane) were separated by 10–12% sodium dodecyl sulfate-polyacrylamide (SDS-PAGE) and transferred onto polyvinylidene fluoride (PVDF) membranes. Membranes were blocked with 5% non-fat milk for 1 h at room temperature, followed by overnight incubation at 4 °C with primary antibodies: CD9 (1:2000, Proteintech, #20597-1-AP), TSG101 (1:1000, Proteintech, #28283-1-AP), and Calnexin (1:5000, Proteintech, #10427-2-AP). After washing, membranes were incubated with HRP-conjugated goat anti-rabbit IgG secondary antibody (1:5000, Proteintech, #B900210) for 1 h at room temperature. Finally, images were captured with the gel imaging system.

### 2.8 RNA isolation and analysis

Total RNA was extracted using the miRNeasy Mini Kit (Qiagen, #217004) according to the manufacturer’s instructions. Briefly, exosome suspensions were lysed in QIAzol reagent, followed by chloroform extraction and column purification. RNA was eluted in 30–50 μL RNase-free water. The RNA concentration and purity were measured using a NanoDrop 2000 spectrophotometer (Thermo Fisher Scientific), and samples with an A260/A280 ratio between 1.8 and 2.1 were used for subsequent analyses. RNA integrity and potential genomic DNA contamination were assessed by 1.5% agarose gel electrophoresis, and RNA quality was further verified using a Qsep100 Bio-Fragment Analyzer (BiOptic).

### 2.9 Small RNA library preparation and sequencing

Small RNA libraries were constructed from ≥5 ng of glycosylated exosomal RNA using the NEBNext® Small RNA Library Prep Set for Illumina® (NEB, #E7330) according to the manufacturer’s instructions. The libraries were amplified (12–15 PCR cycles), size-selected (~140–160 bp) using the E-Gel Power Snap system, and assessed for quality with a Qsep100 Bio-Fragment Analyzer and Qubit 4.0 fluorometer. Pooled libraries were sequenced on the Illumina NextSeq 550 platform with single-end 50-bp reads.

### 2.10 Small RNA sequencing data analysis

The raw data from the Illumina NextSeq 550 sequencing system were converted to fastq format. The 3′ adapter (with no mismatches) was then trimmed using cutadapt v1.18. Trim Galore was used to further trim low-quality sequences with a base quality score below 20. Non-coding RNAs, other than miRNA, were filtered using the rfam and ensembl databases, followed by additional filtration of exon mRNA. miRNA identification and quantification were conducted using miRDeep2 v2.0.1.3. The quantification of miRNA expression levels was converted to reads per million (RPM) and shown with log2 (RPM+1). Differential expression of miRNAs across distinct groups was assessed by permutation tests, with significance set at *p* < 0.05.

### 2.11 Quantification of serum glycosylated exosomal miRNAs by RT-qPCR

Reverse transcription was performed using the miRcute Plus miRNA First-Strand cDNA Kit (Tiangen Biotech Co. Ltd., catalog #KR211), followed by real-time PCR using the miRcute Plus miRNA qPCR Kit (SYBR Green) (Tiangen Biotech Co. Ltd., catalog #FP411). miR-4429 and miR-20a were selected as endogenous references for the analysis of WGA- and LCA-exosomal miRNAs, respectively, while cel-miR-39 was chosen as an exogenous reference for total exosomal miRNA detection by the UC method. The 2^−ΔΔCt method was employed for quantification. The primers are shown in the Supplementary material Table S2.

### 2.12 Statistical analysis

GraphPad Prism 9.3.0 and SPSS 27.0 were used to analyze the experimental data. Group comparisons were conducted with a non-parametric Mann–Whitney *U* test, with significance set at *p* < 0.05. The miRNA panel was established using binary logistic regression.

### 2.13 Bioinformatics analysis

Target genes for these glycosylated exosomal miRNAs were first predicted with the PITA database. mRNA expression levels were then analyzed via the GEPIA2 database. The visualization of predicted results was achieved using Gephi 0.9.3. Finally, the Gene Ontology (GO) functional annotation and Kyoto Encyclopedia of Genes and Genomes (KEGG) pathway enrichment analysis of the target genes were performed using the R software package.

## 3 Results

### 3.1 WGA- and LCA-coated magnetic beads can selectively enrich tumor-derived exosomes

**Figure 1:**
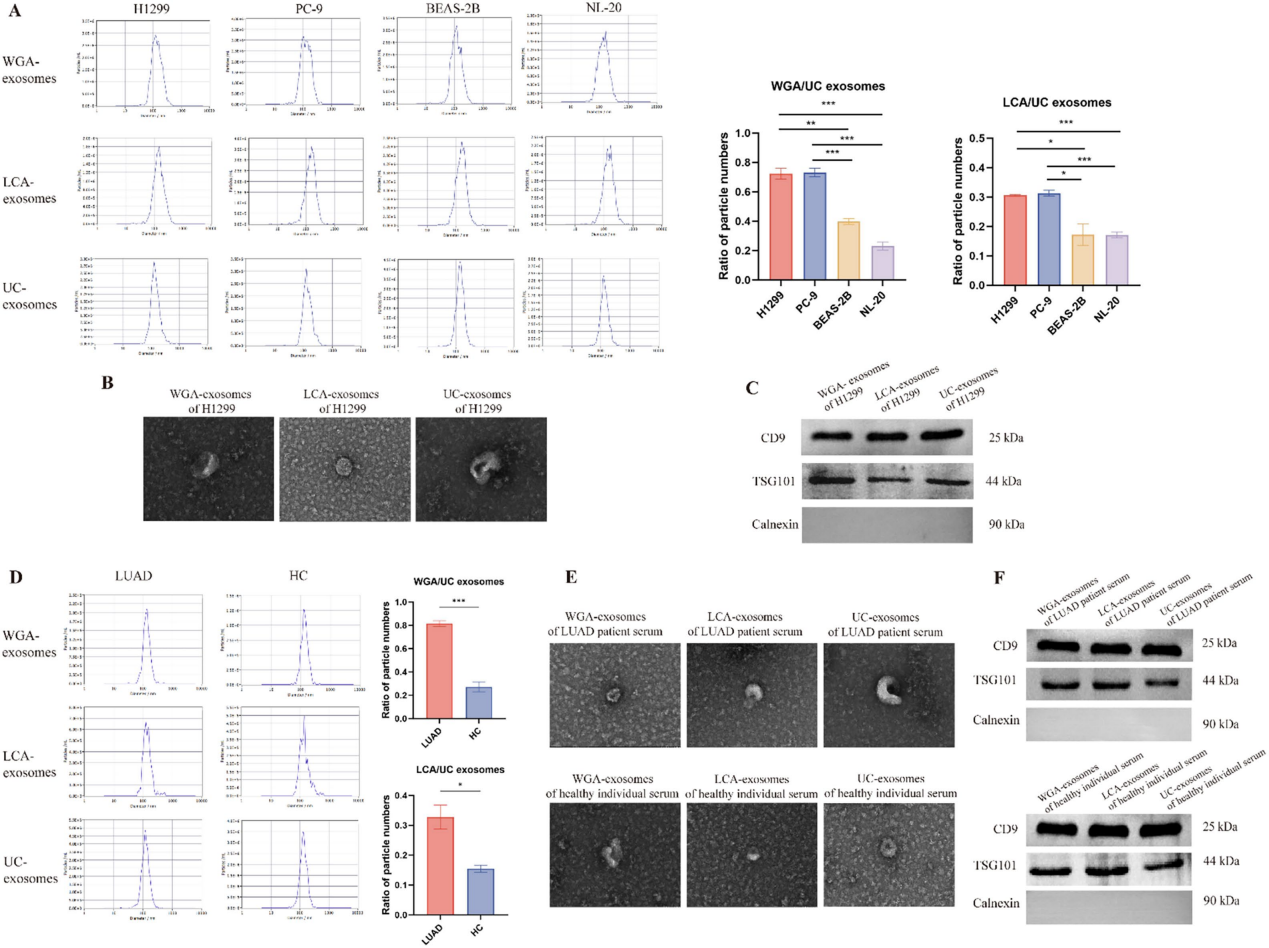
Identification of cell-derived and serum-derived exosomes isolated through the GlyExo-Capture approach. **(A)** NTA analysis of exosomes isolated using WGA-coated beads, LCA-coated beads, and the UC method, comparing the glycosylated exosome capture efficiency across different cell lines. *, **, and *** indicate *p* < 0.05, *p* < 0.01, and *p* < 0.001, respectively. **(B)** TEM images showing the morphology of H1299-derived exosomes; scale bar: 100 nm. **(C)** WB analysis revealed the expression levels of typical exosomal markers, such as TSG-101 and CD9 (positive in exosomes), and Calnexin (negative in exosomes), in H1299-derived exosomes. **(D)** NTA analysis of serum exosomes isolated using WGA-coated beads, LCA-coated beads, and the UC method to compare the efficiency of glycosylated exosome capture from the sera of LUAD patients and healthy individuals. *, *** indicate *p* < 0.05, *p* < 0.001, respectively. **(E)** TEM images showing the morphology of serum-derived exosomes; scale bar: 100 nm. **(F)** WB analysis of serum-derived exosomes.

To demonstrate the enrichment capability of WGA- and LCA-coated magnetic beads for LUAD-derived exosomes, we initially employed WGA- and LCA-coated magnetic beads, as well as the conventional UC method, to isolate exosomes from the CM of two LUAD cell lines (PC-9 and H1299) and two normal cell lines (BEAS-2B and NL-20), respectively. Subsequently, NTA was conducted to quantify and characterize the isolated exosomes. We considered the exosomes isolated by UC to represent the total exosomes. To compare the isolation efficiency of different methods, we calculated the exosome yield as the ratio of particle numbers obtained from WGA- or LCA-based enrichment to those from UC. The results showed markedly higher ratios of particle numbers (WGA-exosomes/UC exosomes; LCA-exosomes/UC exosomes) in LUAD cells compared to normal cells (Figure 1A), indicating that WGA- and LCA-coated magnetic beads can capture more tumor-derived exosomes. The concentrations of exosomes obtained using different isolation methods are detailed in Supplementary material Table S3. We further characterized exosomes isolated from the CM of H1299 cells using three methods, through TEM and WB. The TEM results showed that WGA- and LCA-exosomes, like UC-exosomes, possessed distinct membranous structures, with typical cup-shaped or spherical morphologies (Figure 1B). WB confirmed the presence of the exosomal markers CD9 and TSG101 and the absence of Calnexin, an endoplasmic reticulum-associated protein. The lack of Calnexin expression indicates minimal contamination from intracellular organelles, confirming the purity of the isolated exosomes (Figure 1C). Finally, we isolated exosomes from the sera of LUAD patients and healthy individuals using WGA- and LCA-coated beads, with UC-exosomes serving as controls. Our NTA results showed that the WGA- and LCA-coated magnetic beads captured a higher relative concentration of glycosylated exosomes in the sera of LUAD patients compared to healthy individuals, highlighting the capability of the GlyExo-Capture approach to enrich tumor-derived exosomes (Figure 1D; Supplementary material Table S4). TEM and WB results confirmed the reliability of WGA- and LCA-coated magnetic beads in isolating exosomes from serum (Figures 1E,F). Therefore, this GlyExo-Capture approach could be utilized for the detection of miRNA in serum WGA-exosomes and LCA-exosomes, enabling the investigation of their early diagnostic value for LUAD.

### 3.2 Small RNA sequencing revealed candidate WGA-exosomal miRNAs

**Figure 2:**
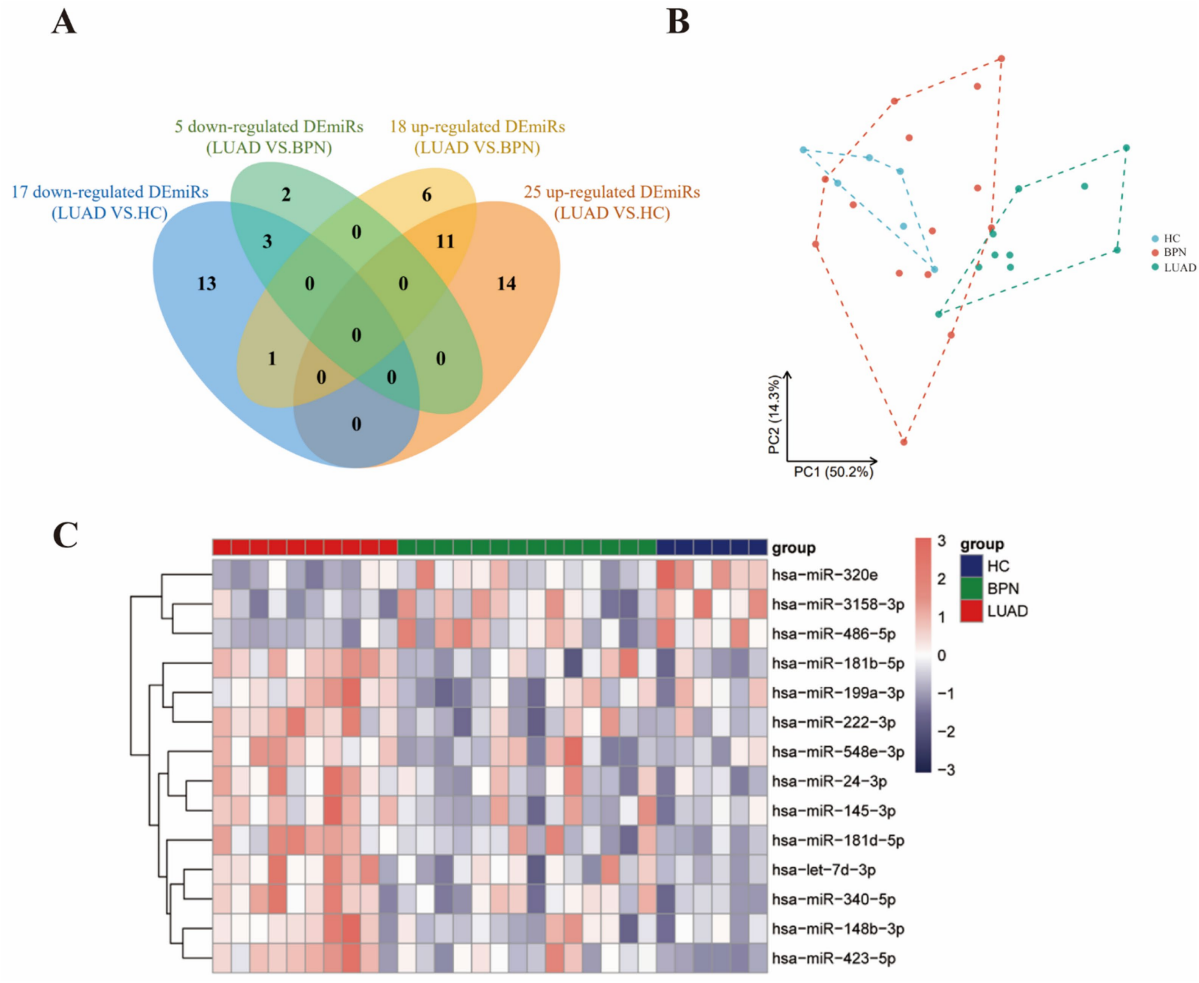
Small RNA sequencing of WGA-exosomal miRNAs in sera from LUAD patients, BPN patients, and HCs. **(A)** The Venn diagram showed the overlapping and non-overlapping DEmiRs. **(B)** PCA including the 14 overlapping DEmiRs. **(C)** Heatmap of 30 samples in the screening set.

To identify serum WGA-exosomal miRNAs for early diagnosis of LUAD, WGA-exosomes isolated from the sera of 10 early-stage LUAD patients, 14 BPN patients, and 6 HCs were analyzed for small RNA sequencing. The results showed 42 differentially expressed miRNAs (DEmiRs) of LUADs compared with HCs, and 23 DEmiRs compared with BPNs (*p* < 0.05). There were a total of 14 overlapping DEmiRs exhibiting similar trends, including 11 upregulated DEmiRs and 3 downregulated DemiRs (Table 1; Figure 2A). Principal component analysis (PCA) showed that DEmiRs exhibit a certain degree of discrimination between LUADs and normal controls (NCs) (NCs: BPNs and HCs) (Figure 2B), and the heatmap displayed the average expression levels of these overlapping DEmiRs in each sample (Figure 2C).

**Table 1:** Overlapping DEmiRs between LUAD vs. BPN and LUAD vs. HC (*p* < 0.05).

miRNA ID	LUAD vs. BPN		LUAD vs. HC	
	Fold change	*p*-value	Fold change	*p*-value
hsa-miR-320e	0.650222921	0.04	0.374933736	0
hsa-miR-3158-3p	0.637219214	0.04	0.457104804	0
hsa-miR-486-5p	0.68241682	0.022	0.585648961	0
hsa-miR-199a-3p	1.426443634	0.003	1.22642869	0.048
hsa-miR-222-3p	1.477727573	0.003	1.447556665	0.01
hsa-miR-148b-3p	1.33045605	0.042	1.501298874	0.038
hsa-let-7d-3p	1.352668884	0.024	1.566478603	0.004
hsa-miR-423-5p	1.217372297	0.036	1.620860552	0.004
hsa-miR-181b-5p	1.354543777	0.01	1.691941579	0
hsa-miR-24-3p	1.409606809	0.022	1.76311583	0.014
hsa-miR-145-3p	1.616343664	0.025	1.921450945	0.007
hsa-miR-548e-3p	1.812409714	0.033	2.044205421	0.008
hsa-miR-340-5p	1.421823451	0.041	2.208717311	0.007
hsa-miR-181d-5p	1.674475939	0.027	2.894927449	0.006

### 3.3 Candidate WGA- and LCA-exosomal miRNAs were identified to establish the predictive panel

**Figure 3:**
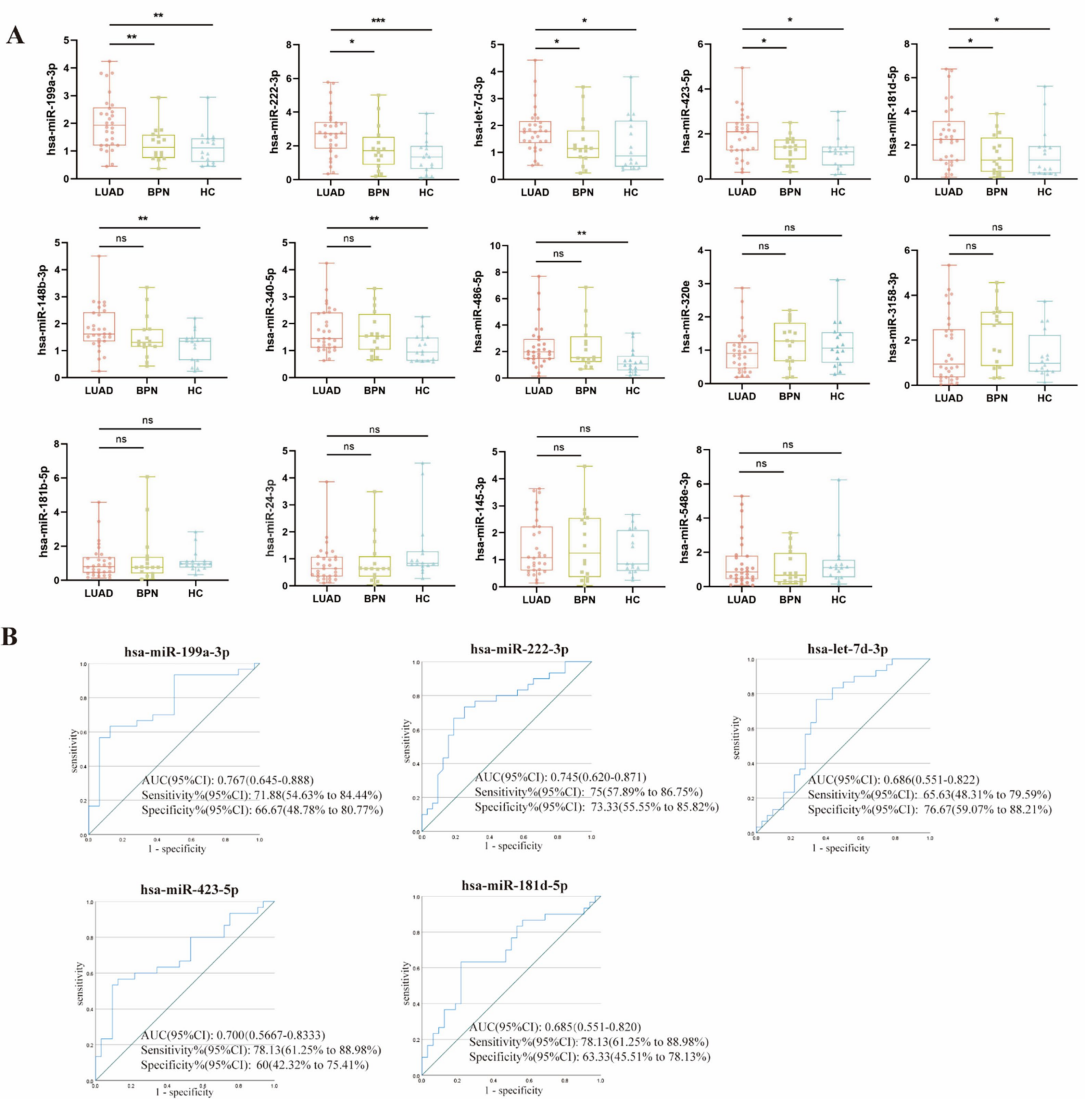
Confirmation of the serum candidate WGA-exosomal miRNAs. **(A)** Expression levels of the 14 WGA-exosomal miRNAs. *, **, *** indicate *p* < 0.05, *p* < 0.01, *p* < 0.001, respectively. **(B)** ROC curve analysis of the five WGA-exosomal miRNAs that are differentially expressed between LUAD vs. BPN and LUAD vs. HC (*p* < 0.05) as determined by RT-qPCR.

Based on the results of small RNA sequencing, an independent set was established to verify the 14 overlapping WGA-exosomal DemiRs, which comprised 30 early-stage LUAD samples and 32 NC samples (16 HCs and 16 BPNs). As shown in small RNA sequencing, miR-4429 was stably and abundantly expressed in all samples, making it a suitable endogenous reference. RT-qPCR analysis confirmed its stability, with a coefficient of variation (CV) of 0.95%, further supporting its suitability as an internal control. RT-qPCR results showed that the expression levels of five WGA-exosomal DEmiRs (miR-199a-3p, miR-222-3p, let-7d-3p, miR-423-5p, and miR-181d-5p) in LUADs were notably elevated compared to those in BPNs and HCs (Figure 3A), consistent with the small RNA sequencing results. Additionally, compared to those in the HC group, the levels of miR-148b-3p, miR-340-5p, and miR-486-5p in the LUAD group exhibited marked upregulation, while there was no significant difference with those in the BPN group. However, miR-320e, miR-3158-3p, miR-181b-5p, miR-24-3p, miR-145-3p, and miR-548e-3p did not exhibit any significant differences. Furthermore, receiver operating characteristic (ROC) curves and area under the curves (AUC) were used to assess the diagnostic performance of the five above WGA-exosomal DEmiRs (miR-199a-3p, miR-222-3p, let-7d-3p, miR-423-5p, and miR-181d-5p) in distinguishing LUAD patients from NCs. Notably, the AUC values of miR-199a-3p and miR-222-3p were the highest, at 0.767 and 0.745, respectively, with both sensitivity and specificity exceeding 65% (Figure 3B), indicating their ability of effectively distinguish early-stage LUAD patients from NCs.

**Figure 4:**
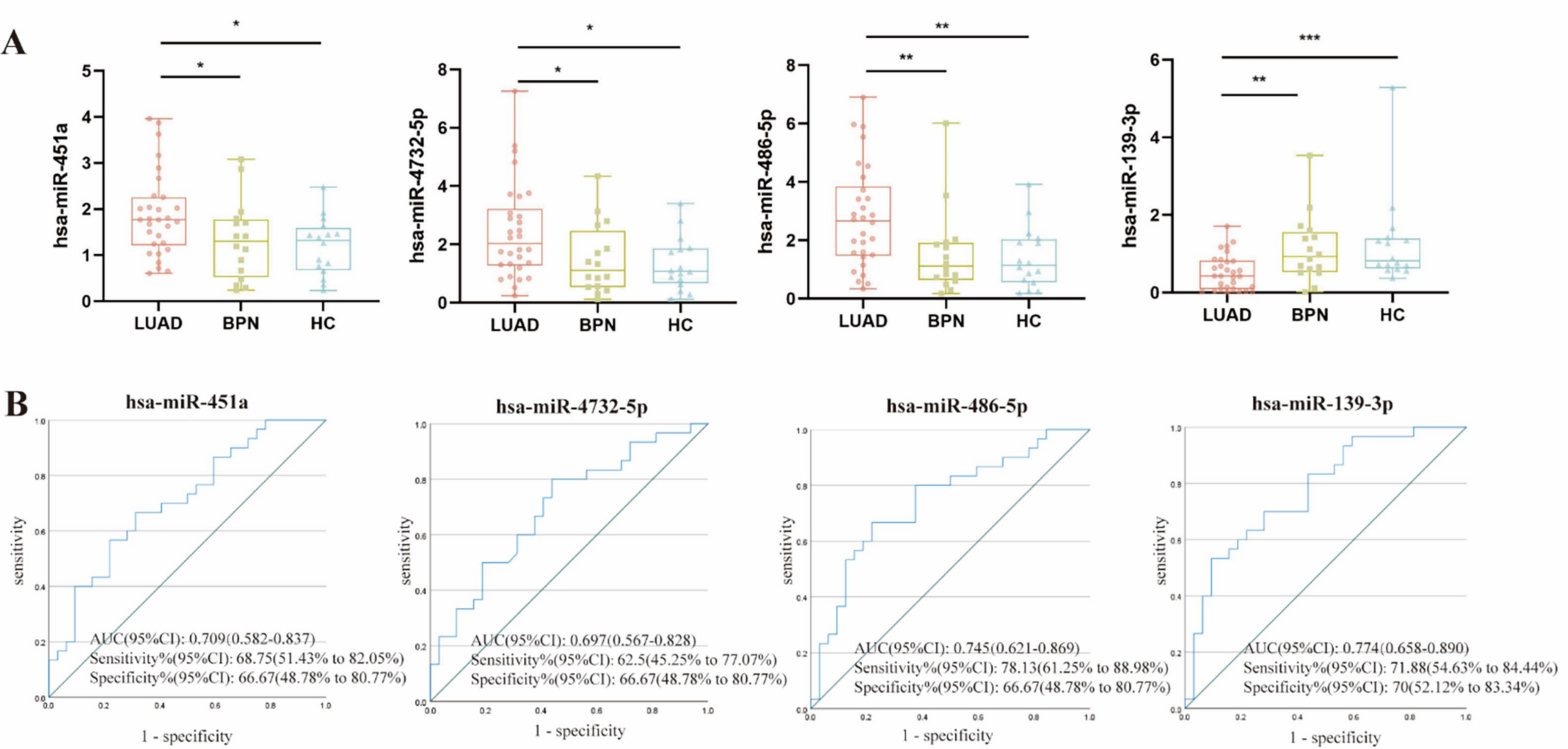
Confirmation of the serum candidate LCA-exosomal miRNAs. **(A)** Expression levels of the 4 LCA-exosomal miRNAs. *, **, *** indicate *p <* 0.05, *p* < 0.01, *p* < 0.001, respectively. **(B)** ROC curve analysis of the four LCA-exosomal miRNAs.

Our pilot study established an LCA-exosomal miRNA panel comprising miR-451a, miR-4732-5p, miR-486-5p, and miR-139-3p for the early diagnosis of LUAD (24). In this study, miR-20a, which was used as the internal control in our previous research, was further validated by RT-qPCR, confirming a CV of 1.02%, indicating its stability as a reference gene for LCA-exosomal miRNAs. To further develop an optimal diagnostic panel combining serum WGA- and LCA-exosomal miRNAs for early-stage LUAD, we proceeded to validate these four serum LCA-exosomal miRNAs using the aforementioned 30 early-stage LUADs and 32 NCs (16 HCs and 16 BPNs). In comparison to HCs and BPNs, early-stage LUAD patients exhibited marked elevation of serum LCA-exosomal miR-451a, miR-4732-5p, and miR-486-5p, but a significant decline of serum LCA-exosomal miR-139-3p (Figure 4A). Meanwhile, the ROC curves indicated that the AUC values of miR-486-5p and miR-139-3p were the highest at 0.745 and 0.774, respectively, with both sensitivity and specificity more than 65% (Figure 4B).

**Figure 5:**
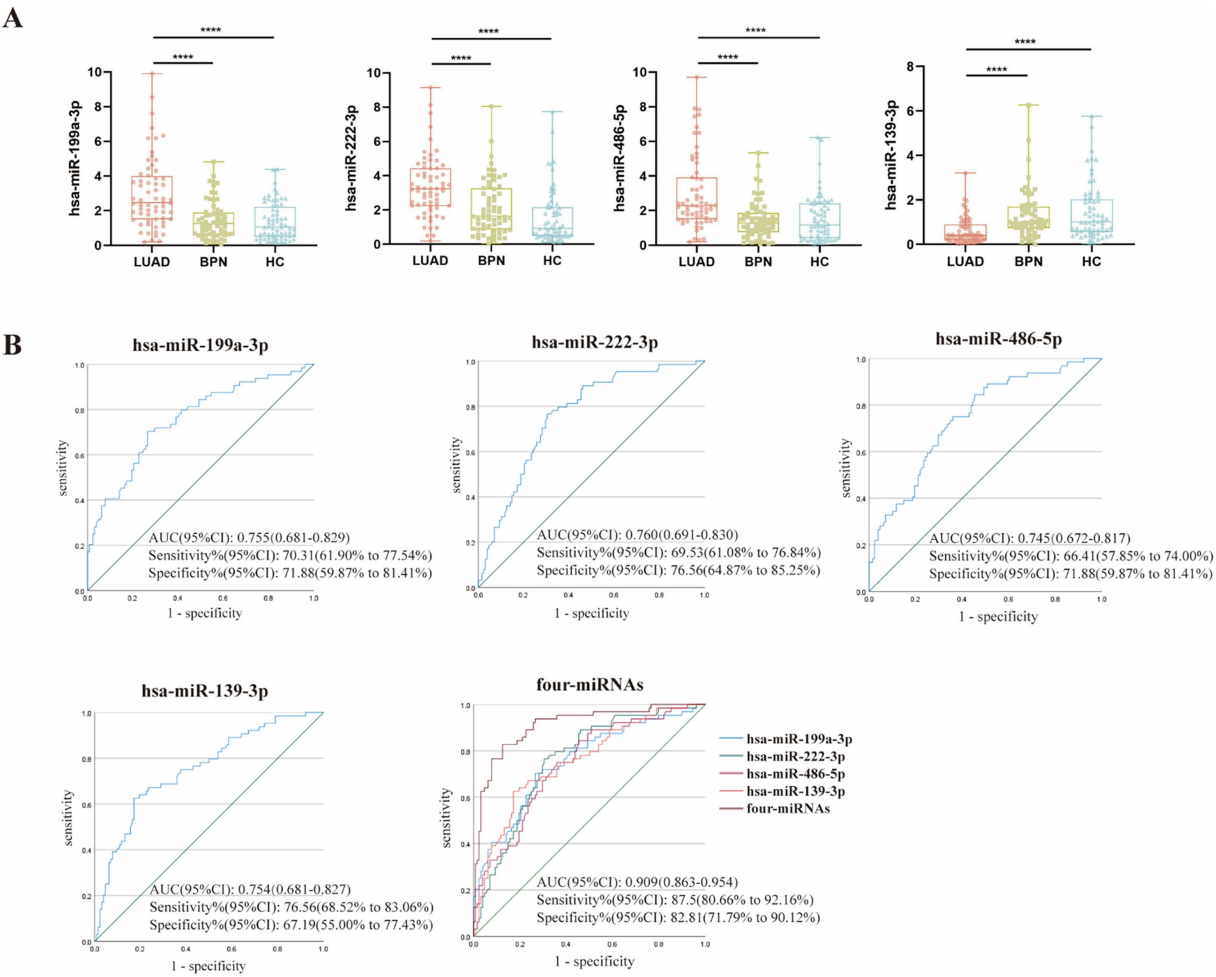
Expression levels of serum WGA-exosomal miRNAs (hsa-miR-199a-3p, hsa-miR-222-3p) and LCA-exosomal miRNAs (hsa-miR-486-5p, hsa-miR-139-3p) in the training set, and the diagnostic potential of the 4-miRNA panel in distinguishing LUAD patients from NCs. **(A)** Expression levels of the four miRNAs. ****indicates *p* < 0.0001. **(B)** ROC curve analysis for each of the four miRNAs and the 4-miRNA panel.

Next, we sought to establish a predictive miRNA panel, such as serum WGA-exosomal miR-199a-3p and miR-222-3p, as well as serum LCA-exosomal miR-486-5p and miR-139-3p. These four miRNAs were evaluated by an additional 192 serum samples, comprising 64 early-stage LUADs and 128 NCs (64 HCs and 64 BPNs). The results showed increased levels of serum WGA-exosomal miR-199a-3p, miR-222-3p, as well as serum LCA-exosomal miR-486-5p, along with decreased levels of serum LCA-exosomal miR-139-3p in LUADs compared with those in BPNs and HCs (Figure 5A). At the same time, we conducted ROC analysis for each individual miRNA and its combination. The results showed that the four miRNAs exhibited moderate efficacy in diagnosing early-stage LUAD (AUC = 0.755, 0.760, 0.745, and 0.754). Furthermore, we analyzed the 4-miRNA panel by a binary logistic regression model, yielding the formula: Logit(P) = 0.568 × miR-199a-3p + 0.304 ×  miR-222-3p + 0.772 ×  miR-486-5p - 1.437 × miR-139-3p - 2.907. Notably, the ROC analysis indicated that the combination of WGA- and LCA-exosomal miRNAs had significantly superior diagnostic efficacy in distinguishing early LUAD patients from NCs with an AUC of 0.909 at 87.5% sensitivity and 82.81% specificity compared to that of four individual miRNAs (Figure 5B).

### 3.4 The diagnostic value of the 4-miRNA panel

**Figure 6:**
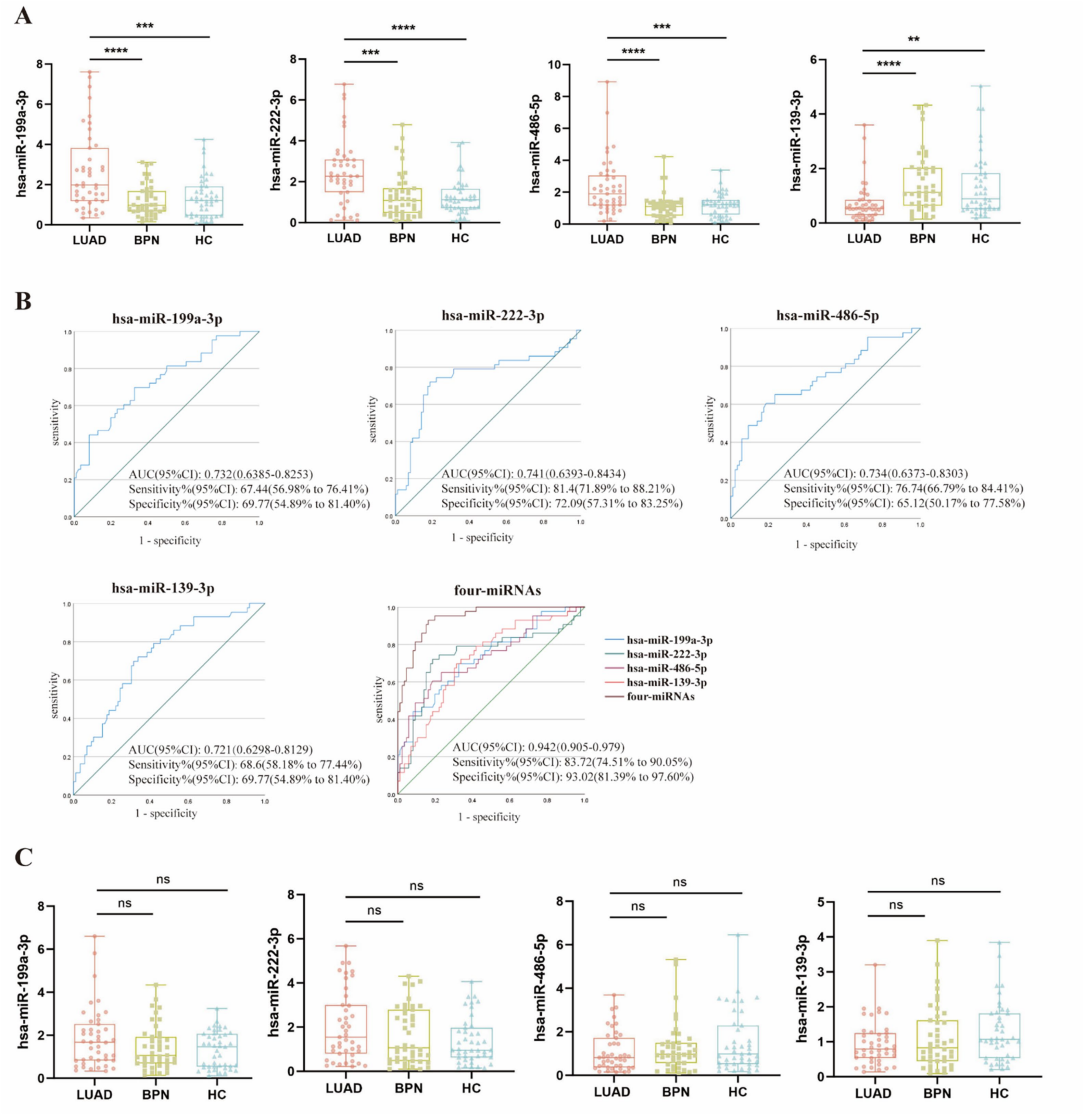
Expression levels of serum WGA-exosomal miRNAs (hsa-miR-199a-3p, hsa-miR-222-3p) and LCA-exosomal miRNAs (hsa-miR-486-5p, hsa-miR-139-3p) in the validation set, and the diagnostic performance of the 4-miRNA panel in distinguishing between LUAD patients and NCs. **(A)** Expression levels of the four miRNAs. **, ***, ****indicate *p* < 0.01, *p* < 0.001, *p* < 0.0001, respectively. **(B)** ROC curve analysis for each of the four miRNAs and the 4-miRNA panel. **(C)** The expression levels of the four miRNAs in total serum exosomes isolated using the UC method.

We further determined the diagnostic value of the combination of serum WGA- and LCA-exosomal miRNAs for early-stage LUAD patients by an independent validation set comprising 43 early-stage LUAD patients and 86 NCs (43 HCs and 43 BPNs), which was consistent with that in the training set (Figure 6A). Subsequently, the binary logistic regression model was also performed in the validation set, yielding the formula: Logit(P) = 0.755 ×  miR-199a-3p + 1.062 ×  miR-222-3p + 2.222 ×  miR-486-5p − 2.320 × miR-139-3p − 5, and the 4-miRNA panel was shown to present an AUC of 0.942 at 83.72% sensitivity and 93.02% specificity, higher than each of four miRNAs (Figure 6B), determining the optimal diagnostic value of this 4-miRNA panel for early-stage LUAD patients. Additionally, four serum total exosomal miRNAs isolated using the UC method on the same samples from the validation set did not show significant differences among the LUADs, BPNs, and HCs (Figure 6C).

### 3.5 Target gene prediction and functional enrichment analysis of candidate DEmiRs

**Figure 7:**
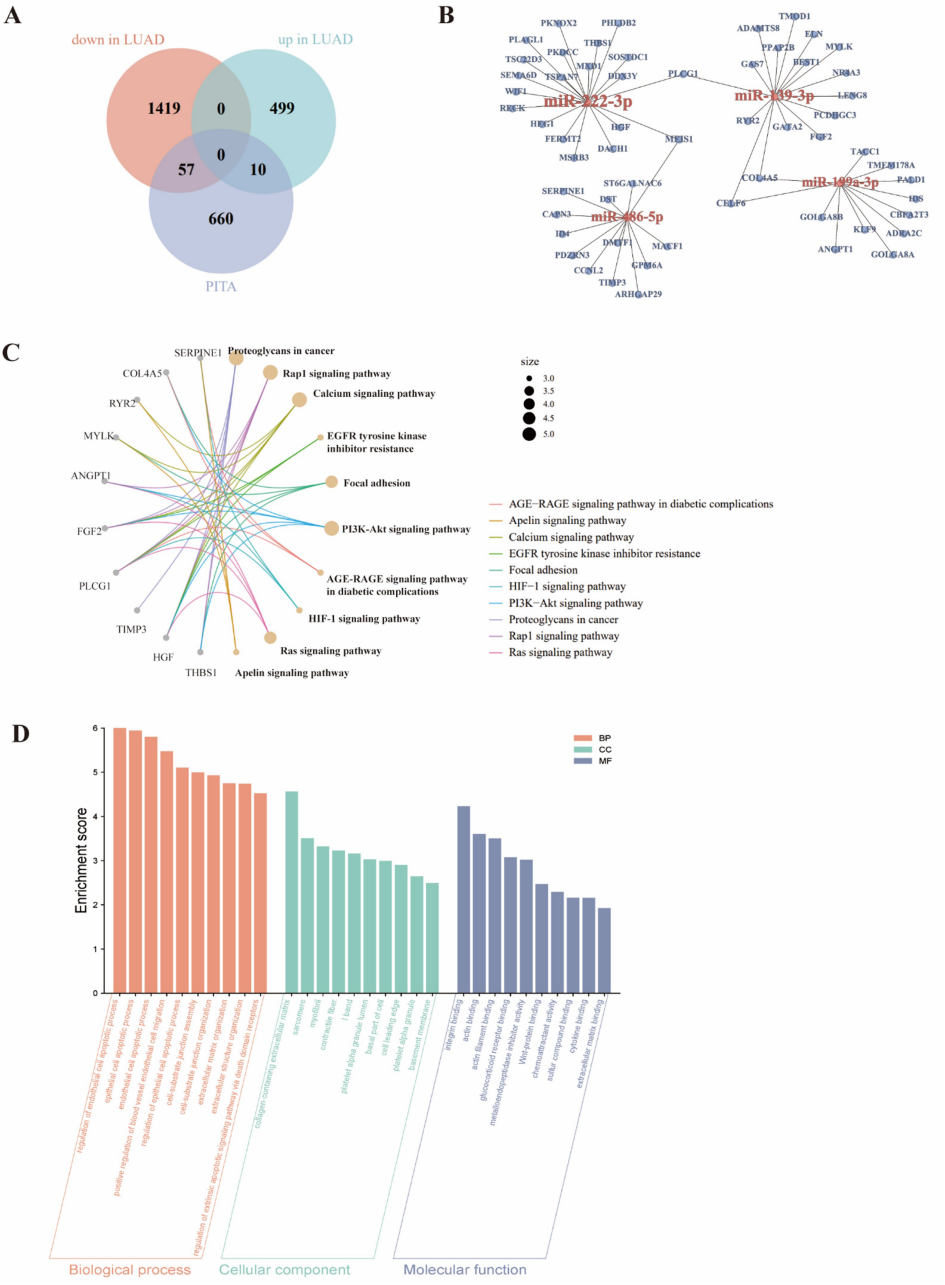
Target gene prediction and pathway analysis of the 4 miRNAs screened in our study. **(A)** Screening target genes using the PITA and GEPIA2 databases. **(B)** Interaction networks of the four miRNAs and their target genes. **(C)** KEGG pathway analysis for target genes functional evaluation of the four miRNAs. **(D)** The GO enrichment results of target genes across biological processes, cellular components, and molecular functions.

Especially mentioned, four miRNAs have been documented to be closely associated with the phenotypic characteristics of lung cancer (25–30). To illustrate the biological functions of these four miRNAs, we used the Platform for Integrating Target Accessibility (PITA) database to predict a total of 727 target genes. To determine the mRNA expression levels of 727 target genes, we further screened the downregulated mRNAs in LUAD samples using GEPIA2 (31). The analysis revealed 1985 differentially expressed genes in LUAD samples, with 1,476 downregulated and 509 upregulated genes. Importantly, among the 1,476 downregulated genes, 57 were predicted targets of the four miRNAs (Figures 7A,B). Next, we identified the potential functions of these target genes through KEGG (32) pathway enrichment and GO (33) functional annotation. The data retrieved from the KEGG indicated that these genes are enriched in signaling pathways closely associated with LUAD, including proteoglycans in cancer, Rap1 signaling pathway, calcium signaling pathway, EGFR tyrosine kinase inhibitor resistance, focal adhesion, and PI3K-Akt signaling pathway (Figure 7C). Furthermore, the results of GO analysis indicated that these target genes are associated with functions relevant to tumor development, such as positive regulation of blood vessel endothelial cell migration, epithelial cell apoptotic process, collagen-containing extracellular matrix, basal part of cell, integrin binding, glucocorticoid receptor binding, etc. (Figure 7D). These results suggested that the four tumor-derived exosomal miRNAs might be responsible for the regulation of LUAD development.

## 4 Discussion

Currently, circulating tumor DNA (ctDNA), free miRNAs, and multi-omics panels have been widely studied for early LUAD detection. However, ctDNA is present in low concentrations in the bloodstream (34), free miRNAs degrade easily, and multi-omics approaches face cost and feasibility challenges (35). In contrast, tumor-derived exosomal miRNAs are actively secreted by tumor cells, protected by lipid bilayers, and closely reflect the tumor microenvironment, making them promising non-invasive biomarkers. Therefore, efficient enrichment of these miRNAs is key to advancing early LUAD diagnosis.

Traditional extraction methods, such as UC, size exclusion chromatography, and filtration, are limited in distinguishing tumor-derived exosomes (36–38). In contrast, immunoaffinity enrichment techniques, such as lectin-based methods, offer promising alternatives (39, 40). Recent advances have demonstrated that lectin-based strategies enable not only EV capture but also detailed profiling of EV surface glycomes, and in some cases, the classification of glycan-defined EV subpopulations. For example, Saito et al. applied a 96-lectin microarray to characterize EV glycomes and successfully distinguished EVs originating from different cell sources (41). Kanao et al. (42) established a lectin-immobilized spongy monolith affinity chromatography system that classified EVs based on surface glycan structures and revealed distinct EV subpopulations through proteomic comparison. In this study, leveraging WGA’s preference for GlcNAc-containing glycans, including limited recognition of sialylated motifs, and LCA’s specificity for core-fucosylated N-glycans, we successfully enriched LUAD-derived exosomes using the GlyExo-Capture approach with WGA- and LCA-conjugated beads. Compared to conventional exosome isolation methods, GlyExo-Capture significantly reduces processing time to just 15 min and simplifies sample processing. It eliminates the need for specialized equipment and requires only a small serum volume, making it well-suited for clinical applications while minimizing patient burden. Moreover, it efficiently enriches tumor-derived exosomes while preserving sample integrity, further supporting its clinical applicability. It is worth noting that the GlyExo-Capture approach is designed to preferentially enrich tumor-derived exosomes rather than to isolate a completely tumor-exclusive population. This tumor-biased enrichment is supported by both our cell-based experiments and analyses of patient serum. Nevertheless, we did not directly define the precise cellular origin of these glycosylated exosomes, and a proportion of vesicles may still originate from non-malignant cells. Future studies incorporating single-vesicle analysis, co-detection of established tumor markers, and mass-spectrometry-based glycoproteomic profiling will be critical to dissect the heterogeneity of lectin-captured exosomes and to further refine glycan-based enrichment strategies for more specific capture of tumor-derived vesicles.

In this study, we analyzed miRNAs from exosomes isolated by the GlyExo-Capture approach across a cohort of 413 serum samples, comprising screening (*n* = 30), training (*n* = 254), and validation (*n* = 129) sets. Through small RNA sequencing and RT-qPCR, we successfully identified and validated a panel of four serum glycosylated exosomal miRNAs, including WGA-exosomal miR-199a-3p and miR-222-3p, as well as LCA-exosomal miR-486-5p and miR-139-3p, which effectively distinguish LUAD patients at an early stage from BPNs and HCs. In the training and validation sets, the AUC of the 4-miRNA panel for early LUAD patients was shown to be as high as 0.909 and 0.942, respectively. The use of multiple independent cohorts strengthens the clinical significance and generalizability of the model, providing compelling evidence for its diagnostic utility.

Further bioinformatics analysis revealed that the target genes of these four miRNAs are closely associated with signaling pathways responsible for tumorigenesis, with significant enrichment of tumor-related functions and biological processes, suggesting that tumor cells might regulate LUAD progression by secreting tumor-associated miRNAs via exosomes. Additionally, these four miRNAs have been reported to play regulatory roles in various cancers, including breast, gastric, and bladder cancer (43–46), with particular relevance to lung cancer. Notably, miR-199a-3p functions as a tumor suppressor by targeting Rheb and enhances sensitivity to gefitinib (27), while miR-222-3p promotes malignant traits and gemcitabine resistance by targeting SOCS3 (47). miR-486-5p has also been suggested to act as a tumor suppressor by targeting ARHGAP5 and PIK3R1, thereby facilitating NSCLC progression (29, 30). Similarly, miR-139-3p exerts tumor-suppressive effects in LUAD by targeting TRIP13 (26). Interestingly, despite their established tumor-suppressive roles in NSCLC, miR-199a-3p and miR-486-5p exhibited a marked increase in serum tumor-derived glycosylated exosomes in this study. The “exosomal escape” hypothesis (24) revealed that tumor cells could release certain tumor suppressive molecules through exosomes to maintain their malignant characteristics. This is also supported by the observation of suppressive miR-4732-3p being discarded from NSCLC cells into the bloodstream via selective sorting by hnRNPK to fucosylated exosomes, leading to a significant elevation in the level of serum LCA-exosomal miR-4732-3p in our pilot study (20). As a result, whether LUAD cells prevent the suppressive effects of miR-199a-3p and miR-486-5p by “exosome escape” is worth exploring.

Collectively, using the efficient GlyExo-Capture approach to isolate glycosylated exosomes, we provide the first depiction of both WGA-exosomal miRNAs and LCA-exosomal miRNAs in the sera of LUAD patients and successfully developed an optimal 4-miRNA panel combining two serum WGA-exosomal miRNAs and two serum LCA-exosomal miRNAs for early diagnosis of LUAD. These results pave the way for early detection of LUAD through the analysis of serum glycosylated exosomal miRNAs. Building on this approach, our panel could be explored for integration with low-dose computed tomography to improve decision-making in cases of indeterminate pulmonary nodules. Additionally, a multi-marker strategy, such as combining protein biomarkers or other molecular signatures, might further enhance specificity.

Admittedly, this study has several potential limitations. First, the demographic characteristics of the study population may affect the generalizability of our findings, requiring further validation in larger multi-center cohorts to confirm the robustness of the 4-miRNA panel across diverse genetic backgrounds. Additionally, its prognostic relevance remains to be explored, particularly in predicting disease progression and treatment response. Third, further studies are needed to better characterize the origin and heterogeneity of WGA- and LCA-exosomes using additional validation approaches, as well as to explore a broader range of glycan-binding molecules (e.g., MAL, AAL, or ConA), thereby enabling more comprehensive mapping of exosomal surface glycosylation and further optimization of glycan-based strategies for tumor-biased exosome enrichment. Finally, investigating whether these four miRNAs are involved in tumor glycosylation pathways and elucidating the mechanisms underlying their selective sorting into glycosylated exosomes could provide deeper insights into LUAD biology and potential therapeutic implications.

## Data Availability

The datasets presented in this study can be found in online repositories. The names of the repository/repositories and accession number(s) can be found at: https://www.ncbi.nlm.nih.gov/, PRJNA1151848.

## Glossary

LUAD: lung adenocarcinoma
miRNAs: microRNAs
GlcNAc: N-acetylglucosamine
WGA: wheat germ agglutinin
LCA: lentil lectin
EV: extracellular vesicle
UC: ultracentrifugation
MBL: magnetic bead solution
WBL: wash buffer
EBL: elution buffer
PBS: phosphate buffered saline
TEM: transmission electron microscopy
NTA: nanoparticle tracking analysis
WB: Western Blot
CM: conditioned media
DEmiRs: Differentially Expressed miRNAs
BPN: benign pulmonary nodule
HC: healthy control
PCA: principal component analysis
NC: normal control
CV: coefficient of variation
ns: no significance
ROC: receiver operating characteristic
AUC: area under the curves
KEGG: Kyoto Encyclopedia of Genes and Genomes
GO: Gene Ontology
ctDNA: circulating tumor DNA
PITA: Platform for Integrating Target Accessibility
