# Supplementary material for: Development of serum glycosylated exosomal microRNAs as biomarkers for early diagnosis of lung adenocarcinoma
Source: Front Med (Lausanne). 2025 Dec 10;12:1695874. doi: 10.3389/fmed.2025.1695874 (PMC12727614; doi:10.3389/fmed.2025.1695874)
Supplement: Supplementary file 1 [file Data_Sheet_1.PDF]

Supplementary Material

Table S1. Clinical data of study subjects.

	Screening set (n = 30)			Training set (n = 254)			Validating set (n =129)		
**Categories**	LUAD (Stage I)	BPN	HC	LUAD (Stage I)	BPN	HC	LUAD (Stage I)	BPN	HC
**Number**	10	14	6	94	80	80	43	43	43
**Age**	53.40 ± 12.84	54.29 ± 9.699	53.17 ± 12.32	55.20 ± 11.21	53.76 ± 10.21	52.54 ± 9.959	55.23 ± 8.214	52.33 ± 9.172	54.40 ± 8.544
**Gender (%)**									
-Male	5(50)	6(42.86)	2(33.33)	53(56.38)	45(56.25)	38(47.5)	20(46.51)	21(48.84)	24(55.81)
-Female	5(50)	8(57.14)	4(66.67)	41(43.62)	35(43.75)	42(52.5)	23(53.48)	22(51.16)	19(44.19)
**Smoking history (%)**	4(40.00)	5(35.71)	2(33.33)	36(38.30)	33(41.25)	25(31.25)	17(39.53)	15(34.88)	16(37.2)

Table S2. Sequences of the primers for qRT-PCR

Gene Name	Primer Sequence (5' to 3')
hsa-miR-4429	F: TCAAAAGCTGGGCTGAGAGGCG
hsa-miR-320e	F: TAAAGCTGGGTTGAGAAGG
hsa-miR-3158-3p	F: ATTATAAGGGCTTCCTCTCTGCAG
hsa-miR-486-5p	F: ATTATTATCCTGTACTGAGCTGCC
hsa-miR-199a-3p	F: ACAGTAGTCTGCACATTGGTTA
hsa-miR-222-3p	F: CGAGCTACATCTGGCTACTGGGT
hsa-miR-148b-3p	F: TCAGTGCATCACAGAACTTTGT
hsa-let-7d-3p	F: CTATACGACCTGCTGCCTTT
hsa-miR-423-5p	F: AATATGAGGGGCAGAGAGCGA
hsa-miR-181b-5p	F: AACATTCATTGCTGTCGGTG
hsa-miR-24-3p	F: ATATGGCTCAGTTCAGCAGGAA
hsa-miR-145-3p	F: GGATTCCTGGAAATACTGTTCT
hsa-miR-548e-3p	F: CAAAAACTGAGACTACTTTTGCA
hsa-miR-340-5p	F: CGTTATAAAGCAATGAGACTGATT
hsa-miR-181d-5p	F: TAACATTCATTGTTGTCGGTGG
hsa-miR-20a	F: CCGCGTAAAGTGCTTATAGTGCAGGTAG
hsa-miR-451a	F: CCGCGAAACCGTTACCATTACTGAGTT
hsa-miR-4732-5p	F: CTGTAGAGCAGGGAGCAGGAAG
hsa-miR-486-5p	F: CGTCCTGTACTGAGCTGCCC
hsa-miR-139-5p	F: TCTACAGTGCACGTGTCTCCAGT
cel-miR-39	F: UCACCGGGUGUAAAUCAGCUUG

Table S3. Concentrations of cell-derived exosomes isolated using different methods

Cell	Exosome	Original concentration (Particles / mL)			Relative concentrations		
		Sample 1	Sample 2	Sample 3	Sample 1	Sample 2	Sample 3
H1299	WGA-exosomes	1750000000	1500000000	1464285714	0.758333333	0.65	0.761428571
	LCA-exosomes	750000000	750000000	642857143	0.303333333	0.303333333	0.312
	UC-exosomes	2307692308	2307692308	1923076923			
PC-9	WGA-exosomes	1785714286	1714285714	1428571429	0.773809524	0.742857143	0.675324675
	LCA-exosomes	678571429	750000000	678571429	0.294047619	0.325	0.320779221
	UC-exosomes	2307692308	2307692308	2115384615			
BEAS-2B	WGA-exosomes	1821428571	1000000000	1821428571	0.430519481	0.4	0.364285714
	LCA-exosomes	642857143	607142857	607142857	0.151948052	0.242857143	0.121428571
	UC-exosomes	4230769231	2500000000	5000000000			
NL-20	WGA-exosomes	964285714	642857143	964285714	0.227922078	0.185714286	0.278571429
	LCA-exosomes	642857143	642857143	607142857	0.151948052	0.185714286	0.175396825
	UC-exosomes	4230769231	3461538462	3461538462			

Table S4. Concentrations of serum-derived exosomes isolated using different methods

Categories	Exosome	Original concentration (Particles / mL)			Relative concentrations		
		Sample 1	Sample 2	Sample 3	Sample 1	Sample 2	Sample 3
LUAD	WGA-exosomes	7100000000	6900000000	6900000000	0.78888889	0.8625	0.79615385
	LCA-exosomes	2000000000	1900000000	3100000000	0.22222222	0.2375	0.35769231
	UC-exosomes	9000000000	8000000000	8666666667			
HC	WGA-exosomes	3100000000	3100000000	2100000000	0.3875	0.344444	0.252
	LCA-exosomes	1400000000	1200000000	1300000000	0.175	0.133333	0.156
	UC-exosomes	8000000000	9000000000	8333333333			
